# Cytoplasmic fragment of CD147 generated by regulated intramembrane proteolysis contributes to HCC by promoting autophagy

**DOI:** 10.1038/cddis.2017.251

**Published:** 2017-07-13

**Authors:** Bo Wu, Jian Cui, Xiang-Min Yang, Zhen-Yu Liu, Fei Song, Ling Li, Jian-Li Jiang, Zhi-Nan Chen

**Affiliations:** 1 National Translational Science Center for Molecular Medicine, Cell Engineering Research Centre and Department of Cell Biology, State Key Laboratory of Cancer Biology, Fourth Military Medical University, 169 Changle West Road, Xi'an 710032, China

## Abstract

Hepatocellular carcinoma (HCC) is one of the most lethal and prevalent cancers worldwide. CD147 (EMMPRIN or basigin) is a leading gene relating to hepatocarcinogenesis and metastasis, and is detected in transmembrane, exosome or circulating forms in HCC patients. The endosome recycling of CD147 further enhances the function of this oncoprotein from a dynamic perspective. However, previous studies about CD147 mainly focused on one separate form, and little attention has been paid to how the different forms of tumor-derived CD147 changes. Moreover, uncovering the roles of the residual C-terminal portion of CD147 after shedding is inevitable to fully understand CD147 promoting tumor progression. In this study, we discovered that under low-cholesterol condition, CD147 endocytosis is inhibited but its shedding mediated by ADAM10 is enhanced. Further procession of residual CD147 in the lysosome produces nuclear-localized CD147-ICD (intracellular domain of CD147), which contributes to autophagy through NF-*κ*B–TRAIL–caspase8–ATG3 axis. As autophagy endows cancer cells with increased adaptability to chemotherapy, and HAb 18 (a specific antibody targeting CD147) inhibits CD147 shedding and sequential CD147-ICD enhances autophagy, we found the combination of HAb 18 and cisplatin exhibited marked antitumor efficiency.

Transmembrane proteins are of pivotal importance for tumor progression associated with cell adhesion and for inter- and intracellular signaling events.^1, 2^ In response to the microenvironment changing accurately and rapidly, cancer cells have developed two cellular pathways for regulating the level and length of ectodomain and, thus, the function of cell-surface proteins. On one hand, endocytosis removes a considerable number of membrane proteins from the surface of cells.^3, 4^ On the other hand, proteolytic release of transmembrane ectodomains, also termed ectodomain shedding, is an irreversible posttranslational mechanism for liberating their extracellular domain and potentially abolishing or altering their function at the cellular surface.^5^

CD147 (EMMPRIN or basigin) is a tumor-related glycosylated protein that belongs to the immunoglobulin superfamily and exists both in transmembrane and soluble forms.^6, 7^ CD147 expression is increased in many types of tumors, and its elevation is often associated with aggressive disease and poor prognosis.^8^ Hepatocellular carcinoma (HCC) is one of the most lethal and prevalent cancers worldwide.^9^ Classically, transmembrane CD147 contributes to the hallmarks of HCC by participating in carcinogenesis, metabolic reprogramming, epithelial-to-mesenchymal transition (EMT) and chemoresistance.^10, 11, 12, 13, 14^ Although endocytosis and recycling is an important avenue for transmembrane CD147 to enhance cancer progression,^15^ the soluble form that results from the shedding of transmembrane CD147 is also discussed as a marker for hepatocarcinoma.^16, 17^ Even though both forms of CD147 involved in HCC progression have been studied, little attention has been paid to how tumor-derived CD147 modificates in response to changes in the tumor microenvironment, especially the converging point between internalized and soluble CD147. Moreover, uncovering the destiny and roles of the residual C-terminal portion of CD147 after shedding is essential to fully understand the role of CD147 in promoting tumor progression.

In this study, we first demonstrated that cholesterol depletion inhibits the endocytosis of CD147 but that the constitutive shedding of CD147 mediated by ADAM10 is enhanced, which could be inhibited by HAb 18, an antibody targeting CD147 and restraining its accessibility to ADAM10. After shedding, the residual C-terminal of CD147 might undergo a sequential cleavage event in lysosomes, which further produces the nuclear-localized CD147-ICD (intracellular domain of CD147). Moreover, ectopic expression of the CD147-ICD enhances autophagy of HCC cells through the NF-*κ*B–TRAIL–caspase8–ATG3 axis, which certainly favors HCC cell survival under cisplatin treatment. Finally, we confirmed that the combination of HAb 18 and cisplatin exhibited marked antitumor efficiency, suggesting a viable combination therapy with the antibody to overcome tumor drug resistance.

## Results

### Cholesterol depletion inhibits CD147 endocytosis but triggers membrane CD147 shedding

**Figure 1:**
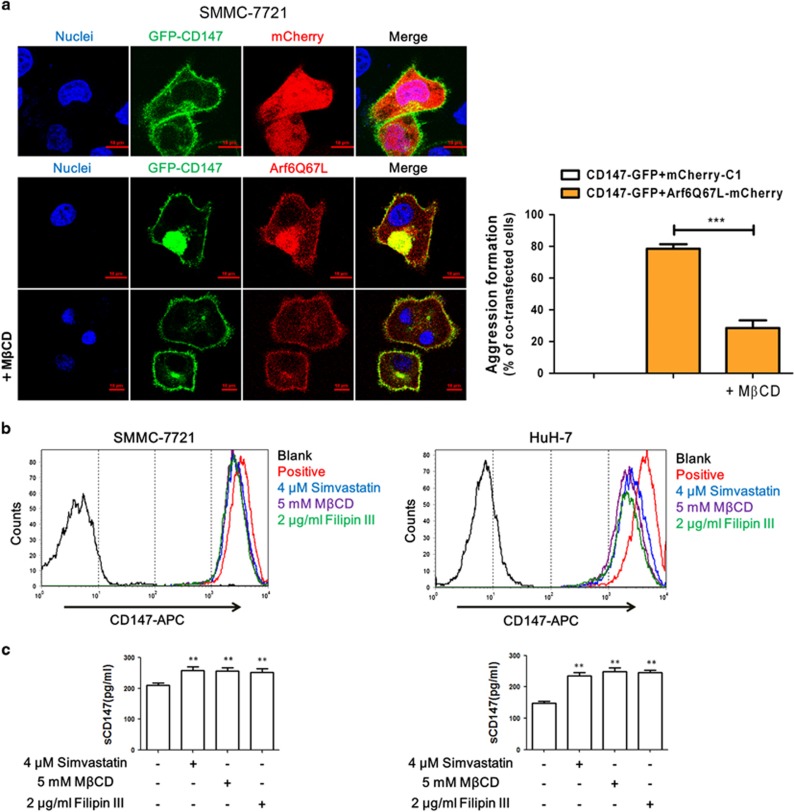
Low cholesterol inhibits CD147 endocytosis but triggers CD147 shedding. (**a**) Representative distribution of exogenous GFP-CD147 co-transfected with mCherry or ArfQ67L-mCherry in SMMC-7721 cells. Growing SMMC-7721 cells were transfected with GFP-CD147 and mCherry/ArfQ67L for 36 h. Exogenous GFP-CD147 was expressed on the membrane but accumulated when ArfQ67L was co-transfected. Treatment with M*β*CD (5 mM) 24 h after transfection significantly inhibited the formation of vacuoles. DAPI staining was used to visualize nuclei. Scale bar, 10 *μ*m. Quantified results are shown as the mean±S.D. of three independent experiments. ***P<0.01. (**b**) Membrane CD147 levels in SMMC-7721 or HuH-7 cells were measured by FACS with anti-CD147-APC after treatment with various cholesterol-depletion materials. (**c**) Soluble CD147 (sCD147) levels in the corresponding culture supernatant of cholesterol-depleted cells were determined by ELISA. **P<0.01

As a tumor-associated protein, CD147 is detected in various forms in tumor patients: transmembrane, exosome, vesicle and circulating forms.^18, 19, 20, 21^ Previously, transmembrane CD147 was reported to internalize through the Arf6-related-CIE (clathrin-independent endocytosis) pathway, while the CIE process is sensitive to cholesterol.^22, 23^ Supplementary Figure S1 demonstrates endocytosis of CD147 in SMMC-7721 cells, and Figure 1a shows that exogenous GFP-CD147 was expressed on the membrane but accumulated in the cytoplasm when cells were co-transfected with Arf6Q67L (red), indicating that Arf6-mediated CD147 endocytosis in SMMC-7721 cells. SMMC-7721 or HuH-7 cells treated with 4 *μ*M simvastatin, 5 mM methyl-*β*-cyclodextrin (M*β*CD) or 2 *μ*g/ml filipin III showed a significant decrease in cellular cholesterol content (Supplementary Figures S2A and B). Treatment with the cholesterol chelator M*β*CD significantly inhibited the formation of the CD147 endocytic vacuoles induced by Arf6Q67L (Figure 1a). However, FACS results showed that the level of membrane CD147 was decreased in cholesterol-depleted SMMC-7721 or HuH-7 cells (Figure 1b). The paradoxical phenomenon was deciphered after corresponding culture supernatants of cholesterol-depleted SMMC-7721 or HuH-7 cells were assessed via ELISA. As shown in Figure 1c, cholesterol depletion resulted in a significant increase of soluble CD147 levels. Indeed, transmembrane CD147 could be cleaved to produce soluble CD147 by MMP-2^24^ or MT1-MMP (MMP-14).^25^ Nonetheless, the expression of these processing enzymes was not sensitive to cholesterol levels (Supplementary Figure S2C). Therefore, cholesterol depletion might contribute to the shedding of membrane CD147 through undefined proteases residing within the membrane or the extracellular space.

### ADAM10 is responsible for the CD147 shedding induced by cholesterol depletion

**Figure 2:**
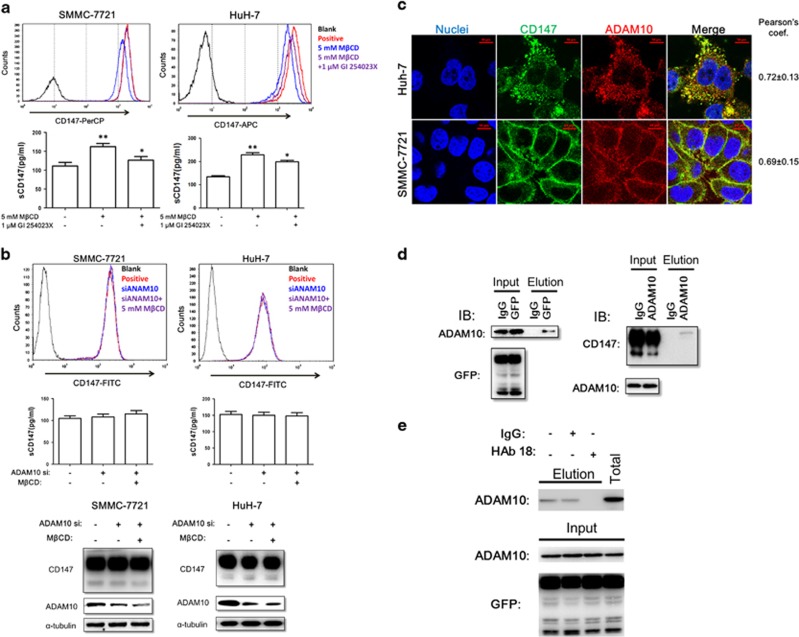
ADAM10 is responsible for CD147 shedding induced by cholesterol depletion. (**a**) Effects of GI254023X on the shedding of CD147. Growing SMMC-7721 or HuH-7 cells were pretreated with 10 *μ*M GI254023X for 30 min before M*β*CD (5 mM) treatment for 4 h. Thereafter, the membrane CD147 of each group was measured by FACS, and sCD147 levels were measured by ELISA. *P<0.05, **P<0.01. (**b**) Knockdown of ADAM10 inhibits M*β*CD-dependent CD147 shedding in SMMC-7721 and HuH-7 cells. Growing SMMC-7721 or HuH-7 cells were first transfected with ADAM10 siRNA for 48 h, and then cells were treated with M*β*CD (5 mM) for 4 h to induce CD147 shedding. Thereafter, membrane CD147 and sCD147 levels were measured as described above. (**c**) Co-localization of CD147 and ADAM10 in SMMC-7721 and HuH-7 cells. The cells (5 × 10^5^) were grown on coverslips for 24 h, fixed and stained with Dylight488-conjugated goat-anti-mouse antibodies (CD147, green) and Dylight594-conjugated goat-anti-rabbit antibodies (ADAM10, red; Pearson’s coefficients are indicated as numerical data to the right of each panel, *n*=3). Bar, 10 *μ*m. (**d**) Co-IP analysis of CD147 and ADAM10. (**e**) HAb 18 blocks ADAM10/CD147 interaction. Resin was immobilized with 4 *μ*g GFP antibody and then cell lysate mixture with/without HAb 18 was added to the resin to analyze ADAM10/CD147 interaction

Studies on the shedding of various membrane proteins have shown that cholesterol depletion triggers the shedding of molecules including APP,^26^ IL-6 receptor^27^ and CD44.^28^ Members of the A disintegrin and metalloprotease (ADAM) gene family are major ectodomain shedding proteinases.^29^ Of the major sheddases of this family, ADAM10 is regulated by the membrane lipid composition and constitutively induces the shedding of membrane CD44,^28^ CX3CL1,^30^ EphA3 receptor^31^ and IL-23R.^32, 33^ To investigate whether ADAM10 participates in low-cholesterol-induced CD147 shedding, we first used GI254023X, an ADAM10-specific inhibitor, to pretreat SMMC-7721 or HuH-7 cells before adding M*β*CD. Then, cholesterol-depleted cells were collected for FACS assay, and the corresponding culture supernatants were applied to ELISA. As shown in Figure 2a, inhibition of ADAM10 significantly suppressed M*β*CD-induced CD147 shedding. Next, we found that cholesterol depletion could not induce CD147 shedding after ADAM10 knockdown by siRNA (Figure 2b). Moreover, the immunofluorescence staining results demonstrated significant co-localization of CD147 with ADAM10 in HCC cells (Figure 2c). The immunoprecipitation results also revealed that CD147 and ADAM10 could form a complex *in vitro* (Figure 2d). Here, cholesterol deprivation from lipid rafts might influence the accessibility of ADAM10 to CD147, as ectodomain cleavage of transmembrane molecules could be controlled by the accessibility of the processing enzyme to the target protein on the membrane (Figure 2e).

Particularly, we found potential CD147-ICD fragments in SMMC-7721 cells stably overexpressing CD147-eGFP (SMMC-7721/CD147-eGFP), and these fragments matched well with ΔECD-eGFP and ICD-eGFP (Supplementary Figure S3). Supplementary Figure S4A shows that treatment of SMMC-7721/eGFP-CD147 with M*β*CD decreased full-length CD147-eGFP levels but increased the levels of ΔECD-eGFP and ICD-eGFP (approximately 30 kDa). As expected, HAb 18, an antibody that targets CD147, inhibited the production of CD147-△ECD in a concentration-dependent manner in SMMC-7721/CD147-eGFP cells (Supplementary Figure S4B). In addition, both SMMC-7721 and HuH-7 cells pretreated with HAb 18 had high levels of membrane CD147 compared with the IgG group (Supplementary Figure S4C). Increased CD147 proteolysis consequently led to accumulation of the circulating form, which is prometastatic to SMMC-7721 and HuH-7 cells (Supplementary Figures S4D and E). Altogether, these results strongly suggest that ADAM10 cleaves transmembrane CD147 under low-cholesterol conditions.

### Residual CD147 was transported to lysosomes for further processing after shedding

**Figure 3:**
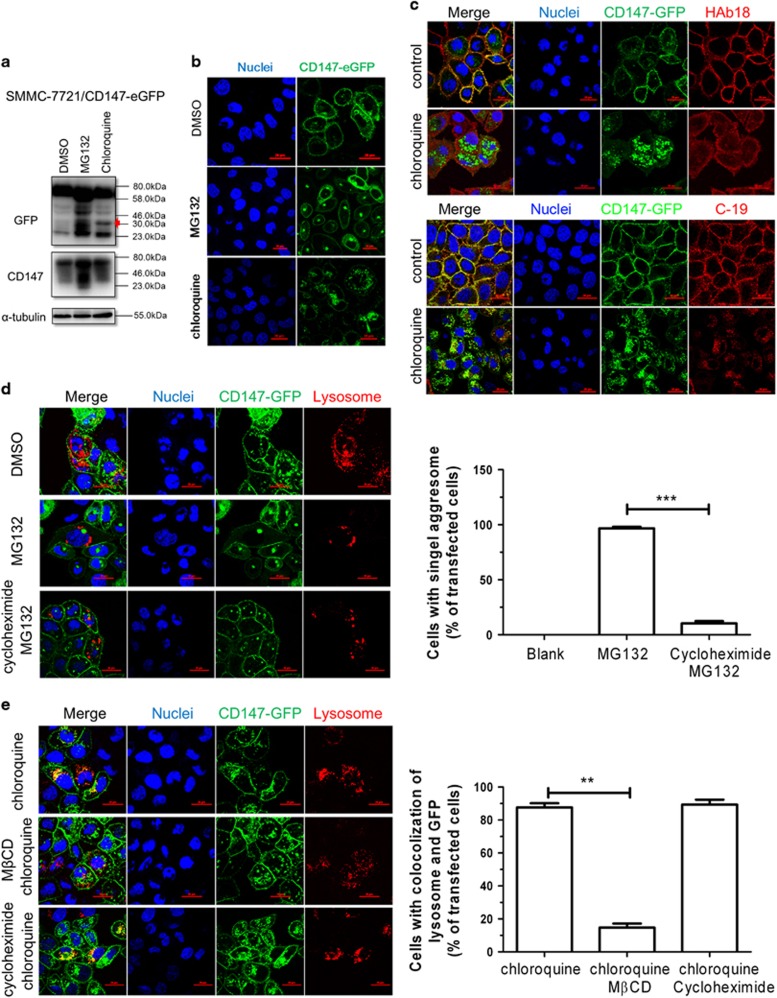
Residual CD147 was transported to lysosomes for further processing. (**a**) The degradation of CD147 follows two simultaneous degradation pathways. Growing SMMC-7721/CD147-GFP cells were first administered 2 *μ*M MG132 or 20 *μ*M chloroquine for 24 h and then assessed via western blot. (**b**) After inhibition of protein degradation with 2 *μ*M MG132 or 20 *μ*M chloroquine, cytoplasm localization of GFP was visualized via confocal microscopy. DAPI staining was used to visualize nuclei. Scale bar, 20 *μ*m. (**c**) Immunofluorescence staining of CD147 (HAb 18: extracellular domain; c-19: intracellular domain) in SMMC-7721/CD147-GFP cells pretreated with 20 *μ*M chloroquine (cells not treated as control). The transfected cells (5 × 10^5^) were grown on coverslips for 24 h and treated with chloroquine (20 *μ*M) for 12 h, and CD147 was then detected by immunofluorescence staining with HAb 18 or c-19, respectively. Bar, 20 *μ*m. (**d**) Distribution of aggresomes induced by MG132 (as indicated by the arrowheads) and lysosomes visualized by CellLight Lysosomes-RFP, BacMam 2.0. Cycloheximide (100 *μ*g/ml) pretreated for 12 h to inhibit protein synthesis in the lower panel. Scale bar: 20 *μ*m. Quantified results are shown as the mean±S.D. of three independent experiments. ***P<0.001. (**e**) Distribution of scattered aggresomes induced by chloroquine (20 *μ*M). Growing SMMC-7721/GFP-CD147 cells were pretreated with 5 *μ*M M*β*CD for 4 h to inhibit protein internalization in the middle panel. Cycloheximide (100 *μ*g/ml) was pretreated as described above in the lower panel. Scale bar, 20 *μ*m. Quantified results are shown as the mean±S.D. of three independent experiments. **P<0.01

As discussed above, CD147 shedding was mediated by ADAM10 under low-cholesterol condition, and soluble CD147-ECD amplifies the prometastatic potential of HCC cells. ADAM10 is an *α*-secretase responsible and prerequisite for the shedding of soluble ectodomain, and for the release of the ICD (intracellular domain) of the receptor to induce cell signaling.^34^ Accordingly, residual CD147 after shedding should have its own destiny. Particularly, intramembrane proteolysis, or the cleavage of proteins within the plane of a membrane, is a widespread phenomenon and contributes to the functional activation of substrates.^35, 36^ Supplementary Figures S3 and S4A show that SMMC-7721/CD147-eGFP naturally produced ΔECD-eGFP and that ICD-eGFP and cholesterol deletion could induce CD147 shedding and the production of CD147-ICD. Specially, we found that the lysosome inhibitor chloroquine, but not the proteasome inhibitor MG132, significantly suppressed the degradation of CD147-ΔECD (Figure 3a, red arrow). Figure 3b shows that MG132-treated SMMC-7721/CD147-eGFP cells produced a specific aggresome structure while chloroquine-treated cells produced scattered aggresomes. CD147-ΔECD is likely to reside in the scattered aggresomes, as it is specifically accumulated after chloroquine treatment (Figure 3a). Thus, we used HAb 18 and c-19 to distinguish the extracellular and intracellular domains of CD147, respectively. As shown in Figure 3c, both HAb 18 and c-19 recognized membrane CD147 under normal conditions. After treatment with chloroquine, scattered aggresomes formed and could only be recognized by c-19. Simultaneously, the control group (cells transfected with eGFP-N1) did not show accumulation after chloroquine treatment (Supplementary Figure S5). These results confirmed that CD147-ΔECD resided in scattered aggresomes induced by chloroquine and that this phenomenon depended on CD147 intrinsically. In addition, we found that the scattered aggresomes induced by chloroquine, but not the specific aggresome induced by MG132, exhibited well co-localization with lysosome (Figure 3e). This indicated that CD147-ΔECD is blocked in lysosomes after chloroquine treatment. As misfolded, CD147 is degraded through the endoplasmic reticulum-associated degradation (ERAD) pathway by the proteasome system,^37^ pretreatment with cycloheximide, a protein synthesis inhibitor, reversed the specific aggresome formation (Figure 3d). However, the scattered aggresomes induced by chloroquine could be partially reversed by M*β*CD but not cycloheximide (Figure 3e). Therefore, residual CD147, that is, CD147-ΔECD, could still internalize and be processed. Together with the former shedding of CD147 mediated by ADAM10, the processing of CD147-ΔECD in the lysosomes discussed here might constitute a novel degradation pathway of CD147. To conclude, after shedding, residual CD147 is transported to lysosomes for further processing.

### Nuclear location of CD147-ICD and ectopic CD147-ICD promotes autophagy of HCC cells

**Figure 4:**
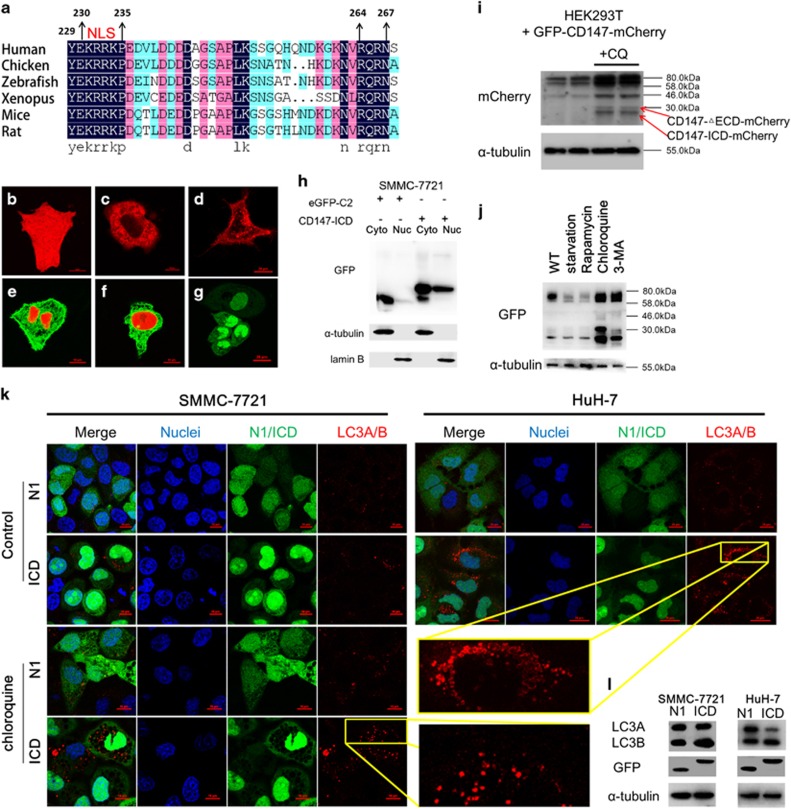
Nuclear-localized CD147-ICD is related to autophagy of HCC cells. (**a**) Sequence alignment of the cytoplasmic tail of CD147 in different species. Identical sequences are highlighted in dark blue, strongly similar sequences in purple and weakly similar sequences in light green. The nuclear leading sequence (NLS) is marked above the sequence. (**b**–**g**) Different constructs were expressed in SMMC-7721 cells, and their locations were visualized via confocal microscopy. (**b**) dsRed2-C1; (**c**) dsRed2-C1-Hook1-S; (**d**) dsRed2-C1-CD147; (**e**) CD147-GFP+dsRed2-C1-CD147-ICD; (**f**) CD147-GFP+dsRed2-C1-CD147-ICD-△264-267; (**g**) GFP-C2-CD147-ICD. Scale bar, (**b**, **c**, **e**, **f**), 10 *μ*m; (**d** and **g**), 20 *μ*m. (**h**) Nuclear accumulation of CD147-ICD in SMMC-7721 cells transfected with GFP-C2-CD147-ICD. SMMC-7721 cells were first transfected with GFP-C2 or GFP-C2-CD147-ICD for 36 h, and then the cytoplasmic and nuclear extracts of the transfected cells were separated with NE-PER nuclear and cytoplasmic extraction reagents (78833, Thermo Scientific). Extracts were applied to western blot analysis, and lamin B was used as a loading control for the nuclear extracts. (**i**) HEK293 cells transfected with GFP-CD147-mCherry were first treated with chloroquine (20 *μ*M) and then assessed via western blot. (**j)** Growing SMMC-7721/CD147-GFP cells were first starved by culturing in EBSS medium for 12 h or administered rapamycin (5 *μ*M), chloroquine (20 *μ*M) or 3-MA (10 mM) for 24 h and then assessed via western blot. (**k**) Immunofluorescence staining of LC3A/B. Scale bar, 10 *μ*m. (**l**) Western blot analysis of LC3A/B in SMMC-7721 (N1/ICD) or HuH-7 (N1/ICD) cells

Many cell-surface transmembrane proteins are subjected to intramembrane proteolysis after ectodomain shedding. At least 14 different intramembrane proteases are known in humans and are found in all the cellular membranes.^36^ As potential CD147-ICD fragments was validated in SMMC-7721/CD147-eGFP, membrane-anchored C-terminal of CD147 will be most likely sequentially processed when transported to lysosomes. Owing to the low level of endogenous CD147-ICD under normal condition, we utilized ectopic CD147-ICD to examine its exact role in the following experiments. Figure 4a shows the sequence alignment of CD147-ICD between different species. A highly conserved region corresponding to a classical nuclear leading sequence (NLS) was found in CD147-ICD. Particularly, CD147-ICD containing the NLS was localized in the nucleus while other controls were not (Figures 4b–g). As shown in Figure 4h, CD147-ICD-GFP was enriched in the nucleus of SMMC-7721 cells transfected with CD147-ICD-GFP. Moreover, we constructed a CD147-expressing vector with its N-terminal fused with GFP and its C-terminal fused with mCherry and transfected the vector into 293 T cells (Supplementary Figure S6). The nuclear accumulation of the mCherry signal revealed regulated intramembrane proteolysis (RIP) of CD147. Taken together, CD147 contains a classical NLS, which can lead CD147-ICD into the nucleus. The predominant nuclear expression of ectopically expressed CD147-ICD might have a direct role in gene regulation.

Even though we observed CD147-ICD in normal condition via different methods, CD147-ICD appears to be unstable, and it is expressed at very low level. It only accumulates when cells are treated with the lysosome inhibitor chloroquine. In GPF-CD147-mCherry transfected 293 T cells, CD147-△ECD-mCherry and CD147-ICD-mCherry were accumulated in the chloroquine-treated group (Figure 4i). Chloroquine is used as an autophagy inhibitor as it allows for the formation and accumulation of autophagosomes but prevents their activity.^38^ Particularly, another autophagy inhibitor 3-MA also induced the accumulation of CD147-△ECD and CD147-ICD, while opposite effect was observed under autophagy-inducing conditions such as starvation or rapamycin treatment (Figure 4j). As chloroquine treatment induced the production and accumulation of CD147-ICD, we decided to examine whether CD147-ICD functions in the autophagy process of HCC cells. Classically, the conversion of LC3A to LC3B monitored by immunoblotting is reflective of autophagic induction. As shown in Figure 4k, increased punctate appearance of LC3 further confirmed that CD147-ICD promoted the autophagy process. Moreover, after transfection of CD147-ICD-GFP, the autophagy maker LC3B is significantly increased in both SMMC-7721 and HuH-7 cells (Figure 4l). To conclude, our results demonstrated the accumulation of CD147-ICD during autophagy inhibition, indicating that CD147-ICD is a product of autophagy. However, the accumulated CD147-ICD in turn promoted autophagy.

### The NF-*κ*B–TRAIL–caspase8–ATG3 axis is involved in CD147-ICD-promoted autophagy of HCC cells

**Figure 5:**
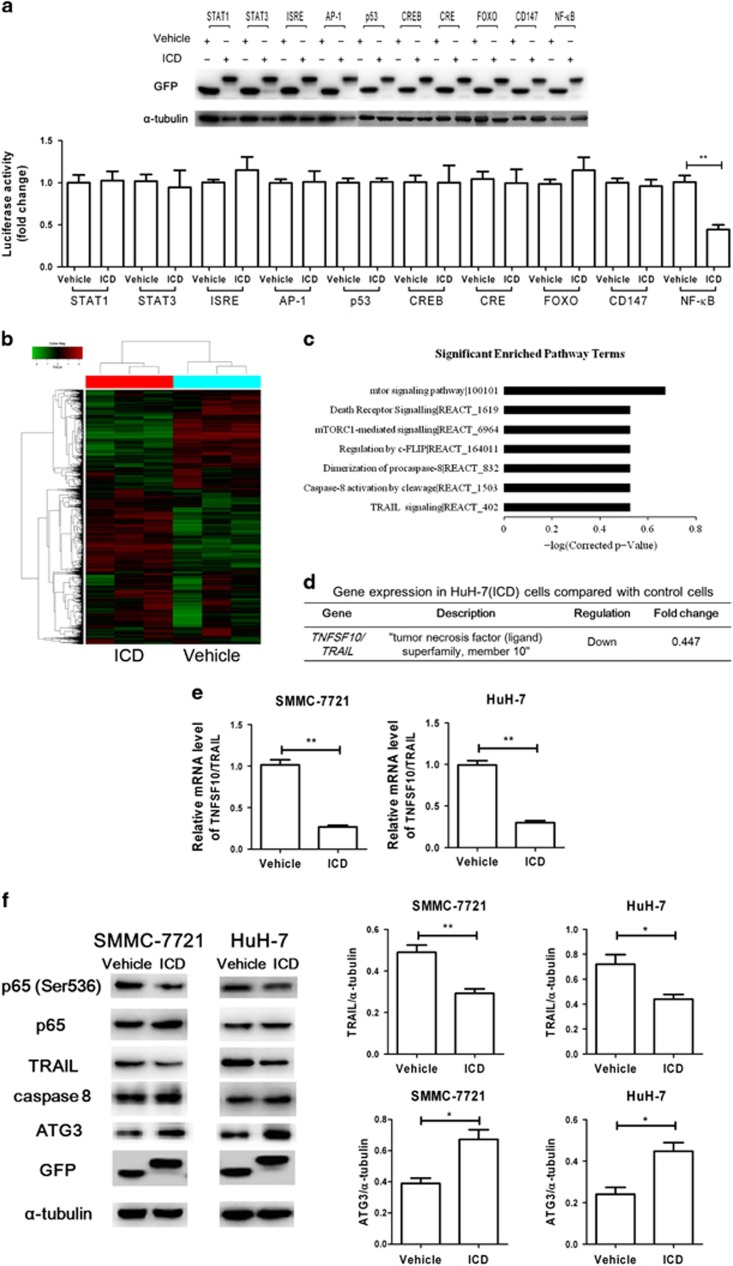
The NF-*κ*B–TRAIL–caspase8–Atg3 axis is involved in the CD147-ICD-promoted autophagy of HCC cells. (**a**) Luciferase assays were carried out in HEK293T cells to measure the effects of a GFP-tagged CD147-ICD (ICD) on the indicated reporters. **P<0.01. (**b**) The cluster heat map shows differentially expressed mRNAs in HuH-7 (GFP) and HuH-7 (CD147-ICD-GFP) cells (*n*=3). Gene expression levels were clustered based on log2 transformation. Red, green, or black represent up-, down-, or normally regulated expression levels, respectively. (**c**) Significant enriched pathway terms. (**d**) Inhibition of TNFSF10/TRAIL in CD147-ICD-overexpressed HuH-7 cells compared to control cells (Vehicle). (**e**) Downregulation of TRAIL mRNA in ICD-transfected SMMC-7721 or HuH-7 cells compared to control (Vehicle). **P<0.01. (**f**)Western blot was applied to analyze the effect of ICD on TRAIL and its downstream caspase8–ATG3 signaling. Western blot scanning densitometry for three independent experiments is shown on the right. Blots were probed for *α*-tubulin to ensure equal protein loading. *P<0.05, **P<0.01

We next analyzed the capability of a GFP-tagged CD147-ICD construct to transactivate a panel of promoter elements, including STAT1, STAT3, interferon stimulated response element (ISRE), activating protein-1 (AP-1), p53, cAMP-response element binding protein (CREB), cAMP-responsive element (CRE), forkhead box O3 (FOXO3), CD147 and NF-*κ*B reporters. CD147-ICD strongly (to 38.3±13.3%) inhibited the NF-*κ*B reporter in HEK293T cells but not the other promoters (Figure 5a). The NF-*κ*B regulatory genes were analyzed by next-generation sequencing (NGS).^39^ To select an effective target downstream of NF-*κ*B signaling, we constructed HuH-7/GFP and HuH-7/CD147-ICD-GFP cells using a lentiviral vector system. Microarray analysis was performed to compare the global differences in gene expression between HuH-7/GFP and HuH-7/CD147-ICD-GFP cells (Figure 5b). Finally, we found that TNFSF10 was regulated by NF-*κ*B and significantly decreased in HuH-7/CD147-ICD-GFP cells (Figures 5c and d). Then, the downregulation of TNFSF10 by CD147-ICD was confirmed by RT-PCR and western blot in both SMMC-7721 and HuH-7 cells (Figures 5e and f). TNFSF10, or TNF-related apoptosis-inducing ligand (TRAIL), elicits tumor-specific cell death through its receptors.^40^ In particular, the cleavage of Atg3 protein by caspase8 was reported to regulate autophagy during receptor-activated cell death.^41^ The results of this study also revealed the accumulation of ATG3 in CD147-ICD-transfected HCC cells (Figure 5f). Therefore, TRAIL downregulation resulting from CD147-ICD overexpression contributes to the enhanced autophagy of HCC cells through the NF-*κ*B–TRAIL–caspase8–ATG3 axis.

### Result 6. CD147-ICD confers chemoresistance to HCC cells through autophagy

**Figure 6:**
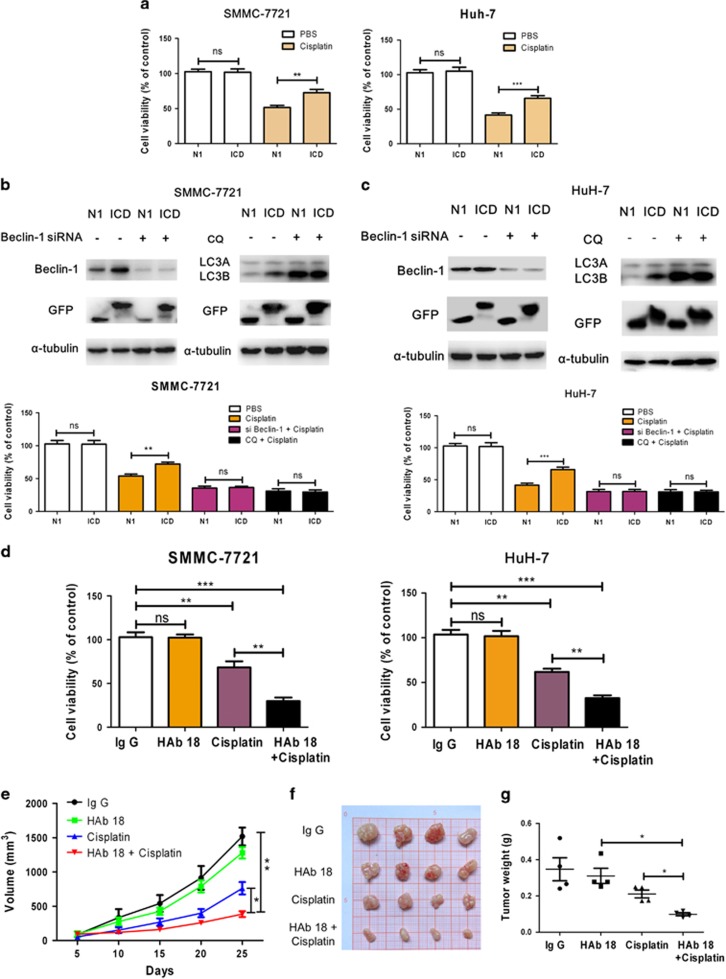
CD147-ICD confers chemoresistance to HCC cells by promoting autophagy. (**a**) SMMC-7721 (N1/ICD) or HuH-7 (N1/ICD) cells were treated with cisplatin (6 *μ*g/ml) for 48 h. Cell viability was determined by MTT assays. Data are presented as the mean±S.E.M., *n*=3. (**b**) Inhibition of autophagy promotes cisplatin-induced cell death in SMMC-7721 (N1/ICD) cells. SMMC-7721 (N1/ICD) cells were transiently transfected with siRNA against beclin-1. The beclin-1 knockdown cells and the control-transfected cells were then treated with cisplatin for 48 h. SMMC-7721 (N1/ICD) cells were simultaneously treated with chloroquine (CQ) to inhibit autophagy and CQ-treated or control cells were then treated with cisplatin for 48 h. Cell viability was assessed by MTT assays. Data are presented as the mean±S.E.M., *n*=3. **P<0.01. (**c**) Inhibition of autophagy promotes cisplatin-induced cell death in HuH-7 (N1/ICD) cells. Huh-7 (N1/ICD) cells were treated as described above. ***P<0.001. (**d**) SMMC-7721 or HuH-7 cells were treated as indicated for 48 h. Cell viability was assayed by MTT assays. Data are presented as the mean±S.D., *n*=3. **P<0.01. ***P<0.001. (**e**) Tumor growth inhibition with HAb 18, cisplatin alone or combined in Balb/c nude mice bearing SMMC-7721 cell line xenografts. Treatment started 5 days after tumor cell inoculation and grew to approximately 100 mm^3^ in size. The mice were treated with intravenous injection of IgG or HAb 18 and/or cisplatin for two cycles of 10 d, *n*=4 tumors per treatment group. The mice were killed on day 25. Tumor size was measured by calipers. Tumor volume was calculated using the formula: (length × width^2^)/2. Data are presented as tumor volume (mm^3^) in the mean±S.D. (**f** and **g**) Primary tumors were harvested and weighed. Each group had four animals, and experiments were repeated twice. Data are expressed as the mean ±S.D. *P<0.05, **P<0.01

The role of autophagy in cancer initiation and treatment has been found to be context-dependent, but it could endow cancer cells with markedly increased adaptability to metabolic crises such as chemotherapy.^42, 43^ Figure 6a shows that CD147-ICD-transfected cells were more resistant to cisplatin treatment based on MTT assay. As CD147-ICD promotes autophagy of HCC cells, we then examined whether CD147-ICD confers chemoresistance to HCC cells through autophagy. Beclin-1 is a crucial protein in the autophagy process. In SMMC-7721 cells, after knocking down beclin-1 by siRNA, cisplatin treatment induced an equal rate of death in both vehicle and CD147-ICD-transfected cells (Figure 6b). Similarly, chloroquine treatment inhibited the final step of the autophagy process. After combined treatment of chloroquine and cisplatin, equal death rates were induced in both vehicle and CD147-ICD-transfected SMMC-7721 cells (Figure 6b). A similar synergistic effect of chloroquine and cisplatin was observed in HuH-7 cells (Figure 6c). Altogether, CD147-ICD confers chemoresistance to HCC cells through autophagy. The shedding of CD147 produced not only the circulating CD147 released to the extracellular environment but also the nuclear-localized CD147-ICD. As HAb 18 inhibited the constitutive shedding process of CD147 mediated by ADAM10, the combined effect of cisplatin and HAb 18 was then examined in HCC cells. As shown in Figure 6d, cisplatin treatment but not HAb 18 treatment for 48 h decreased the viability of HCC cells (to 72.1±8.2% in SMMC-7721 compared to 65.8±4.3% in HuH-7). However, the tumor inhibitory efficacy was increased when cisplatin was combined with HAb 18 to treat HCC cells (to 24.4±4.7% in SMMC-7721 compared to 26.5±3.4% in HuH-7). Moreover, we established a SMMC-7721 tumor xenograft model in nude mice, and Figure 6e shows the tumor progression curve. As shown in Figures 6f and g, the weights of the excised tumors in the control group and HAb 18 treatment group were similar, but both were significantly heavier than that of the cisplatin group, and the combined group corresponded to the most effective results. Finally, the expression of CD147, LC3A/B in tumor tissues collected from mice group with different treatment were compared by Western blot (Supplementary Figure S7). The level of CD147 is slightly increased in HAb 18 treated mice, while LC3B is significantly increased in the group treated with the chemical drug alone. All these results suggested that the antitumor activity of HAb 18 with cisplatin is better than that of HAb 18 alone and indicated that HAb 18 significantly improved the chemosensitivity of SMMC-7721 cells to cisplatin *in vivo*.

## Discussion

Derailed endocytosis and recycling of cell-surface proteins is gradually being recognized as a multifaceted hallmark of malignant cells.^2, 44^ The CIE and recycling of CD147 have critical roles in tumor progression. Ectodomain shedding is an important posttranslational mechanism that increases the range of functions of cell-surface molecules.^45^ Circulating CD147 shed from the cell membrane also enhances tumor progression.^16^ However, the potential relationship between endocytosis and shedding of CD147 has not been previously studied. The cholesterol content of membranes affects endocytosis,^46, 47^ and cholesterol depletion triggers the shedding of these molecules, including APP, IL-6 receptor, CD30 and L1-CAM.^28^ Here, we found that cholesterol depletion inhibits the endocytosis of CD147 but that the constitutive shedding of CD147 mediated by ADAM10 is enhanced. Proteolytically activated ADAMs are responsible for the ectodomain shedding of membrane-associated proteins. Dysregulation of these processes through aberrant ADAM expression or sustained ADAM activity is linked to chronic inflammation, inflammation-associated cancer and tumorigenesis.^5, 29, 33^ ADAM10 activity is regulated by the membrane lipid composition. Ectodomain shedding of CD147 leads to its downregulation on the cell surface and generation of the soluble form with agonistic properties. As the endocytosis and recycling of membrane proteins have vital roles in the progression of cancer, blocking endocytosis of CD147 by depleting cholesterol may be effective in tumor inhibition. However, cholesterol depletion also induces more soluble CD147 released to the extracellular environment, which might make up the function of recycle-blocked CD147 in HCC.

Particularly, we found that, after treating with chloroquine, the ΔECD-eGFP produced in SMMC-7721/CD147-eGFP was accumulated and resided primarily in lysosomes. Intramembrane proteolysis, or the cleavage of proteins within the plane of a membrane, is a widespread phenomenon that contributes to the functional activation of substrates.^36^ Different families of intramembrane proteases have been discovered and characterized.^36^ Although our results did not completely identify the intramembrane protease of CD147, data presented here strongly indicate that the enzyme functions in the normal physiology of lysosomes. In addition, this might constitute a novel pathway of CD147 degradation in addition to endoplasmic reticulum-associated degradation (ERAD) of misfolded CD147.^36, 37^ The functional consequence of intramembrane proteolysis includes activation of membrane-tethered transcription factors and transcriptional activators.^35^ Here, we found the nuclear location of CD147-ICD and its suppressive effect on NF-*κ*B activation. Unstable or of low levels, natural CD147-ICD was not detected in the present study. However, with the fusion vector, we observed the nuclear location of CD147-ICD-mCherry. ADAM10 is actually an *α*-secretase responsible for the signaling of numerous receptors such as Notch and CD44.^29^ For several ADAM substrates, ectodomain shedding is also an initiating and rate-limiting step for subsequent cleavage events. This process is known as regulated intramembrane proteolysis (RIP) and releases intracellular domains that can translocate to the nucleus and regulate gene transcription.^35, 36^ It has been hypothesized that secretase processing of transmembrane proteins may be a cellular housekeeping mechanism to degrade these molecules, as the presence of a transmembrane domain is a barrier to other proteolytic systems.^48, 49^ Therefore, the shedding mediated by ADAM10 may be a prerequisite for CD147-ICD suppression of NF-*κ*B activation.

Autophagy is an evolutionarily conserved self-degradative process that removes damaged proteins and organelles to promote a cell survival response to nutritional starvation or stress conditions.^50, 51^ Overexpressed CD147-ICD promotes autophagy in parallel with an increase in the protein levels of beclin-1 and LC3B and with the accumulation of autophagic vacuoles in the cytoplasm in HCC cells (Figures 4l and 6c). To further investigate the signaling pathways, a microarray analysis was performed, and we found that TRAIL is significantly decreased downstream of NF-*κ*B in CD147-ICD-overexpressing cells. Moreover, it was validated that CD147-ICD promotes autophagy through the TRAIL-caspase8-ATG3 axis (Figure 6). Indeed, CD147 was reported to be involved in autophagy.^52, 53, 54^ In addition to CD147-ICD being a product of autophagy, our results demonstrated a novel pathway by which CD147 contributes to autophagy. The role of autophagy in cancer initiation and treatment has been found to be context-dependent. Autophagy has emerged as a novel cytoprotective mechanism to increase tumor cell survival by escaping chemotherapy-induced cell death. In our study, we found that the cytoprotective function of CD147-ICD to cisplatin disappeared after blocking autophagy with beclin-1 siRNA or chloroquine. Hence, overexpression of CD147-ICD confers chemoresistance to HCC cells through autophagy. Moreover, we demonstrated HAb 18 targeting CD147 combined with cisplatin induced marked antitumor activity. HAb 18 inhibits CD147 shedding and the sequential RIP in lysosomes by blocking the accessibility of ADAM10 to CD147, and these results are in accordance with a previous report that indicated the sensitivity enhancement of the antibody.^55^

**Figure 7:**
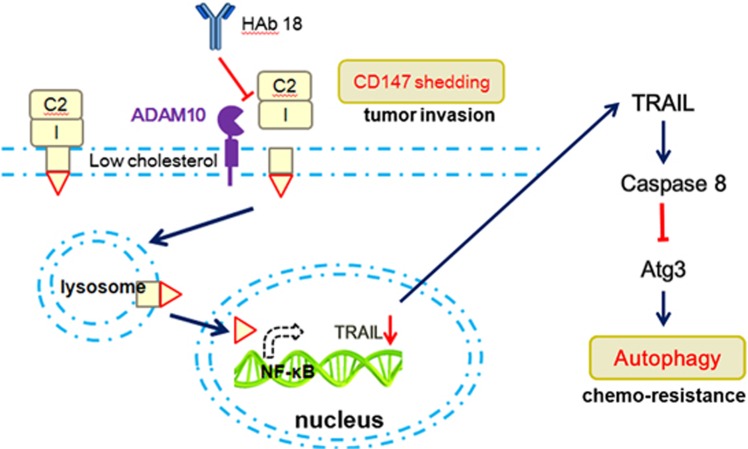
General model for regulated intramembrane proteolysis of CD147 contributes to HCC progression. Under low-cholesterol conditions, constitutive shedding of CD147 mediated by ADAM10 is enhanced. Resulting circulating CD147 promotes malignant phenotypes in HCC cells. Residual CD147 after shedding still undergoes internalization and transports to lysosome for intramembrane proteolysis and releases the nuclear-localized CD147-ICD. Through NF-*κ*B–TRAIL–caspase8–Atg3 axis, CD147-ICD promotes autophagy of HCC cells, the process which certainly benefits cancer cell survival under metabolic crisis (for example, chemotherapy)

To conclude, we describe for the first time that the cholesterol content of the membrane balances the endocytosis and shedding of CD147 and that sequential intramembrane proteolysis produces nuclear-localized CD147-ICD, which modulates autophagy in HCC cells through the NF-*κ*B–TRAIL–caspase8–ATG3 pathway. Moreover, the enhanced tumor chemoresistance exerted by CD147-ICD is mediated in part by augmenting autophagy (concluded in Figure 7). This provides a mechanistic view of previous reports that have demonstrated chemoresistance of CD147 overexpressing cells. Although these findings do not fully elucidate the mechanism of enhanced tumor chemoresistance by CD147-ICD, they suggest potential benefits of combination therapy with antibodies.

## Materials and Methods

### Cell culture

Human SMMC-7721 HCC cells were purchased from the Institute of Biochemistry and Cell Biology (IBCB, Shanghai, China). HEK293T cells were obtained from the American Type Culture Collection (ATCC, Manassas, VA, USA). SMMC-7721 cells and HEK293T cells were cultured in RPMI-1640 (Gibco, New York, NY, USA) with 10% fetal bovine serum, 2 mM glutamine, 100 U/ml penicillin and 100 *μ*g/ml streptomycin in 5% CO_2_ at 37 °C. HuH-7 cells were purchased from the Cell Bank of the JCRB (Tokyo, Japan) and cultured in DMEM medium supplemented with 10% FBS. For studies of amino acid starvation-induced autophagy, cells were cultured in EBSS medium (E2888; Sigma-Aldrich, St. Louis, MO, USA) for 12 h to induce autophagy.

### Plasmids

The following plasmids were used: peGFP-N1 (Clontech, Mountain View, CA, USA), peGFP-C2 (Clontech), pmCherry-N1, dsRed2-C1, pcDNA3.1 (Invitrogen, Carlsbad, CA, USA), eGFP-N1-CD147 (CD147-eGFP; NM_198589), dsRed2-C1-CD147 and pcDNA3.1-Arf6Q67L (NM_001663.3). A sequence encoding eGFP was inserted into the signal peptide of CD147 by overlapping polymerase chain reaction (PCR) and then cloned into peGFP-N1 with *Hind* III/*Not* I to create GFP-CD147.^21^ Then, the intracellular or extracellular domains of CD147 were deleted to generate ΔECD-eGFP and ICD-eGFP, respectively. The sequence coding for the intercellular domain of CD147 (residues 230–269 of CD147) was cloned into peGFP-C2 and dsRed2-C1 to produce eGFP-C2-ICD and dsRed2-C1-CD147-ICD, respectively. RQRNAAs of dsRed2-C1-CD147-ICD were deleted using a QuikChange Lightning Multi Site-Directed Mutagenesis Kit to create dsRed2-C1-CD147-ICD-△264-267. Sequences encoding GFP-CD147 and mCherry were sequentially cloned into pcDNA3.1 with *Xho* I/*Bam* H I and *Bam* H I /*Hind* III to create GFP-CD147-mCherry. Arf6Q67L was cloned into pmCherry-N1 to create pmCherry-N1-Arf6Q67L. The sequence encoding amino acids 486–728 of human Hook1 (NM_015888.4) was cloned into dsRed2-C1 to create dsRed2-C1-Hook1-S. The complete sequences of the above constructs can be obtained upon request.

### Reagents and antibodies

Simvastatin (101314-97-0), filipin III (480-49-9), MG132 (1002628) and cycloheximide (66-81-9) were from Cyman (Michigan, MI, USA). GI254023X (sc-490114) was from Santa Cruz Biotechnology (Dallas, TX, USA). M*β*CD (C4555) and chloroquine (C6628) were from Sigma-Aldrich. Rapamycin (r706203) was from Sangon (Shanghai, China). 3-methyladenine (3-MA) was from Genechem (REVG1007, Shanghai, China). CD147-APC (MA1-10104) and CD147-PerCP (562554) for FACS were from Thermo Fisher Scientific (Pittsburgh, PA, USA) and BD Biosciences (Franklin Lakes, NJ, USA), respectively. Antibodies for mCherry (ab 167453) and TRAIL (ab 65121) were from Abcam (Cambridge, UK); antibodies for GFP (sc-9996) and CD147 (c-19, sc-9754) were from Santa Cruz Biotechnology. Antibodies for ADAM10 (25900-1-AP), lamin B (66095-1-Ig), p65 (10745-1-AP), caspase8 (13423-1-AP) and ATG3 (11262-2-AP) were from Proteintech (Wuhan, China). Antibodies against phospho-NF-*κ*B p65 (Ser536) were from Beyotime (AN371, Nantong, China). LC3 A/B (D3U4C) antibody was from Cell Signaling Technology (Santa Cruz, CA, USA). Antibody for beclin-1 (2026-1) was from Epitomics (Burlingame, CA, USA). IgG for mouse origin (P5000) was from Genia Biotech (Beijing, China). Antibodies for CD147 (HAb 18, IgG1) and *α*-tubulin were developed in our laboratory.^56^ Dylight488- or Dylight594-conjugated secondary antibodies used for FACS or immunofluorescence were from Life Technology (San Jose, CA, USA). Horseradish peroxidase-conjugated secondary antibodies for western blot were from Pierce (Rockford, IL, USA).

#### Construction of cell lines

For SMMC-7721/CD147-eGFP, CD147-eGFP was first transfected into SMMC-7721 using Lipofectamine 2000 (Invitrogen) for 48 h, and the transfected cells were sorted for GFP fluorescence by flow cytometry. The sorted cells were cultured in RPMI-1640 with 10% fetal bovine serum and 1 mg/ml G418 (345811, Calbiotech, Shanghai, China). CD147-ICD overexpression lentiviruses and control vectors were introduced into cells using FuGENE 6 (Roche, Basel, Switzerland). SMMC-7721 (N1/ICD) or HuH-7 (N1/ICD) cell lines were selected by adding 2 *μ*g/ml puromycin to the culture medium. The upregulation of CD147-eGFP/CD147-ICD expression was verified by qPCR, immunoblot and immunofluorescence.

### Modulation of cellular cholesterol and filipin staining

The cells were grown to near confluency in a 24-well plate. The cells undergoing cholesterol depletion were washed twice with serum-free medium and then incubated with 5 mM M*β*CD or 2 *μ*g/ml filipin III for 4 h. For simvastatin experiments, the cells were cultured at 37 °C for at least 24 h in the presence of various concentrations of simvastatin. The reagents were diluted in serum-free RPMI-1640 medium. Cellular cholesterol content was assayed spectrophotometrically using an Amplex Red cholesterol assay kit (Invitrogen).

Filipin staining was carried out with a cholesterol cell-based detection assay kit (10009779, Cyman) according to the manufacturer’s instructions. Briefly, the cells were treated with or without cellular cholesterol modification materials were rinsed with PBS twice and fixed with 1% glutaraldehyde on ice for 15 min. The cells were then rinsed with PBS twice and treated with 50 g/ml filipin for 30 min at room temperature. The cells were rinsed again and examined by UV excitation.

### Soluble CD147 ELISA

The concentration of soluble CD147 in the culture supernatants was determined using a CD147 ELISA kit (R&D Systems, Shanghai, China) according to the manufacturer’s instructions.

### FACS

FACS was performed as previously described.^21^ Briefly, administered cells were detached with PBS containing 0.05% trypsin and incubated with at 4 °C with CD147-APC (1:200, BD Biosciences) in the dark for 30 min and then analyzed using a FACS Arial I analyzer (BD Biosciences) with FCS Express Version 3 software.

### Co-immunoprecipitation of CD147 and ADAM10

The interaction of ADAM10 and CD147 was analyzed by co-immunoprecipitation. A total 1 × 10^6^ SMMC-7721/CD147-eGFP cells were lysed in 100 *μ*l lysis buffer (20 mM Tris-HCl, pH 7.5, 150 mM NaCl, 0.5 mM EDTA, 0.5% NP40 (Tergitol-type NP-40) and a complete protease inhibitor mixture tablet/50 ml buffer) for 1 h at 4 °C under constant agitation and centrifuged at 20 000 g for 20 min. The resulting cell lysate was mixed with 300 *μ*l dilution buffer (20 mM Tris-HCl, pH 7.5, 150 mM NaCl, 0.5 mM EDTA and a complete protease inhibitor mixture tablet/50 ml buffer). For input control, 40 *μ*l of this solution was mixed with 10 *μ*l 5 × loading buffer and incubated for 10 min at 95 °C. AminoLink Plus Coupling Resin (Pierce kit, Lot: 26149), which was immobilized with 4 *μ*g GFP antibody or 10 *μ*g HAb 18 mAb, were washed twice with dilution buffer and subjected to the cell lysate mixture mentioned previously. HAb 18 or IgG (1 *μ*g) was added into cell lysate mixture to block ADAM10/CD147 interaction. Resin and lysate were incubated at 4 °C overnight under constant agitation. Afterwards, the resin was washed three times with PBS, and the eluted samples were collected. Fifty microliters of the resulting supernatant were mixed with loading buffer and incubated for 10 min at 95 °C (referred to elution) for western blotting.

### RNA interference and transfection

The following siRNAs (GenePharma, Shanghai, China) were used in this study. For the siRNA duplex targeting ADAM10,^57^ siRNA and the control nonspecific siRNA were as follows: the sense strand used was 5′-AAAGGAUUCCCAUACUGAC-3′, and the antisense strand was 5′-GUCAGUAUGGGAAUCCUUU-3′ for the siRNA duplex targeting beclin-1,^58^ the sense strand was 5′-CAGTTTGGCACAATCAATA-3′, and the antisense strand was 5′-CAGGAACTCACAGCTCCAT-3′ the control nonspecific siRNA of the sense strand and the antisense strand were 5′-AUCUUGAUCUUCAUUGUGC-3′ and 5′-GCACAAUGAAGAUCAAGAU-3′, respectively. The cells were transfected with the siRNAs or plasmids using Lipofectamine 2000 (Invitrogen).

### Reporter assay

The luciferase reporter for CD147 was kept in our lab.^59^ All the other firefly luciferase reporters (STAT1, STAT3, ISRE, AP-1, p53, CREB, CRE, FOXO3 and NF-*κ*B) were purchased from Transheep (Transheep, Shanghai, China). Twenty-four hours after seeding, the cells were transiently transfected in triplicate with reporters together with pRL-null, a plasmid expressing the enzyme Renilla luciferase, used as an internal control (Promega Corporation). Luciferase assays were carried out according to standard procedures.

### Microarray analysis

For the analysis of the gene expression profiles of HuH-7 (GFP) and HuH-7 (CD147-ICD-GFP) cells, total RNA was prepared. Affymetrix Human U133 Plus 2.0 arrays were used according to the manufacturer’s instructions. The gene expression levels of the samples were normalized and analyzed with Microarray Suite, MicroDB, and Data Mining Tool software (Affymetrix, Santa Clara, CA, USA). Then, the Affymetrix GeneChip Human Genome U133 Plus 2.0 Array was performed at Capital Bio in Beijing.

### RNA isolation, reverse transcription and qPCR

Total RNA was extracted using an Omega R6934-01 Total RNA Kit. cDNA was synthesized using Prime Script RT Reagent (Takara, Dalian, China, DRR037A). qPCR was performed on a LightCycler 2.0 using SYBR Premix Ex Taq (Takara, DRR081A). The results were calculated using the 2^−^^ΔΔ^^Ct^ method.^60^ The following primers were used in this study:

Actin: Forward 5′-TCACCCACACTGTGCCCATCTACGA-3′, Reverse 5′-CAGCGGAACCGCTCATTGCCAATGG-3′ MMP-2: Forward 5′-GCCCATCATCAAGTTCCCCG-3′, Reverse 5′-CCGCATGGTCTCGATGGTAT-3′ MT1-MMP: Forward 5′-GGCGGGTGAGGAATAACCAA-3′, Reverse 5′-GCATCCAGAAGAGAGCAGCA-3′ TNFSF10: Forward 5′-TTGGGACCCCAATGACGAAG-3′, Reverse 5′-TGGTCCCAGTTATGTGAGCTG-3′.

### Chemosensitivity assay by methylthiazolyldiphenyl-tetrazolium bromide (MTT)

The effect of CD147-ICD on HCC cells resistant to cisplatin was assessed by MTT assay, as previously described. Briefly, 5 × 10^3^ cells were seeded in a 96-well plate and cultured for 24 h, and then cisplatin (6 *μ*g/ml) was added to the cells. After an additional 24 h of respective treatments, 20 *μ*l MTT (5 mg/ml) was added to the wells, and the cells were incubated for 4 h. The media were removed and replaced with 100 *μ*l DMSO. The plates were read on a plate reader (Bio-Rad Laboratories, Hercules, CA, USA) at 490 nm, and the reference wavelength was 690 nm. Inhibition of cell growth was measured as the percentage of viable cells relative to the control and calculated as follows: percent viable cells rate=100% × ODT/ODC, where ODT is the average OD value of the treated samples, and ODC is the average OD value of the control samples. The assay was repeated three times. The mean optical density (OD±S.E.M.) was calculated for each group. To investigate the ability of CD147-ICD to promote chemical resistance through autophagy, the cells were transfected with beclin-1 siRNA for 48 h before seeding into a 96-well plate, or cells were treated with CQ (100 nM) to inhibit autophagy.

### *In vivo* antitumor efficacy study

Athymic nude mice (female, 5–6 weeks old) were purchased from Vital River Laboratories (Beijing, China). The animals were housed (five per cage) in specific pathogen-free (SPF) conditions, supplied with food and water ad libitum, and kept on a 12 h light/dark cycle. All the studies were conducted in accordance with approved protocols of the Institutional Animal Care and Use Committee. All the mice were kept in quarantine for 1 week before experimentation. Five mice per subgroup were subcutaneously implanted with 1 × 10^7^ SMMC-7721 cells. When tumors reached a mean volume of 100 mm^3^, the mice were randomized into experimental groups (*n*=4). The mice in different treatment groups received a single dose of IgG (10 mg/kg), HAb 18 (10 mg/kg), cisplatin (3 mg/kg) or HAb 18 combined cisplatin (10 mg/kg HAb 18, 3 mg/kg cisplatin) for two cycles of 10 days. Tumor size was measured every 5 days using digital calipers. The mice were killed via cervical dislocation 25 days after the injection of tumor cells.

### Statistical analysis

All data were expressed as the mean±S.D. and were analyzed using either one-way analysis of variance or two-tailed unpaired Student’s *t*-test. For each parameter of all data presented, *indicates *P*<0.05, **indicates *P*<0.01.
