# Supplementary material for: Cytoplasmic fragment of CD147 generated by regulated intramembrane proteolysis contributes to HCC by promoting autophagy
Source: Cell Death Dis. 2017 Jul 13;8(7):e2925–. doi: 10.1038/cddis.2017.251 (PMC5550841; doi:10.1038/cddis.2017.251)
Supplement: Supplementary Information [file cddis2017251x1.docx]

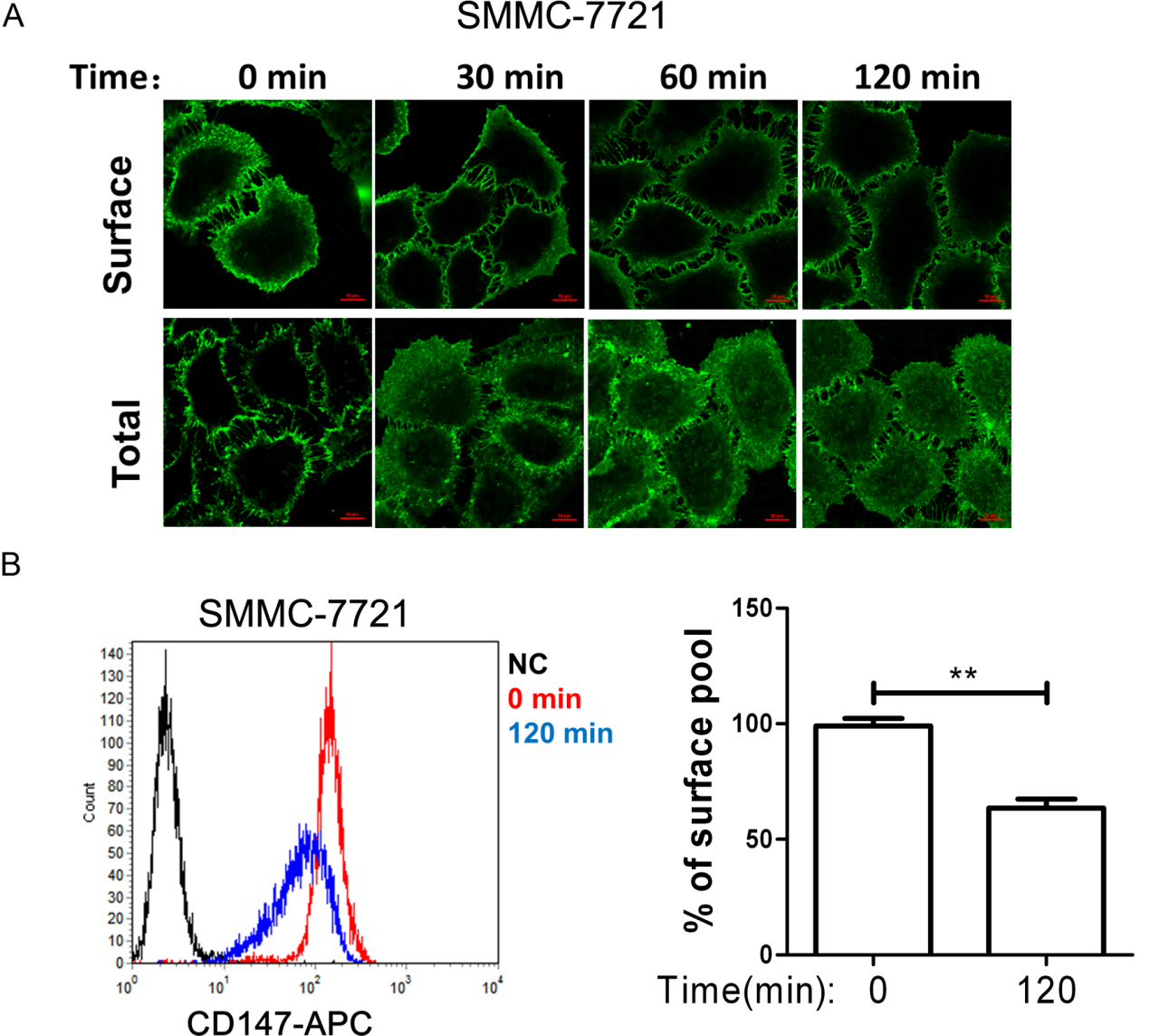
Fig. S1. **Internalization of CD147.** (A) Subconfluent SMMC-7721 cells were washed with ice-cold PBS and incubated with anti-CD147 (HAb 18, 5μg/mL) diluted in RPIM1640 media at 4 °C for 10 min, and then unbounded antibodies were washed. The media was replaced with RPIM1640 media containing 10% fetal bovine serum prewarmed to 37 °C. Cells were allowed to internalize the surface label at 37 °C for indicated periods of time (0-120 min) and were fixed with 4% formaldehyde for 10 min. Samples were then labeled with Dylight-488-conjugated secondary antibody in the dark to immediately identify surface CD147. The lower panel samples were treated with 0.5% Triton X-100 after fixation and labeled with secondary antibodies to view both membrane and cytoplasm (total) CD147. Scale bar=10 μm. (B) After internalization for either 0 min or 120 min as described above, the cells were harvested and washed once with PBS containing 0.5% bovine serum albumin (BSA). Samples were incubated at 4 °C with secondary antibody in the dark for 30 min and then analyzed with FACS. The experiment was performed in triplicate, and the mean and standard error of the resulting data were calculated. **, *p*<0.01.


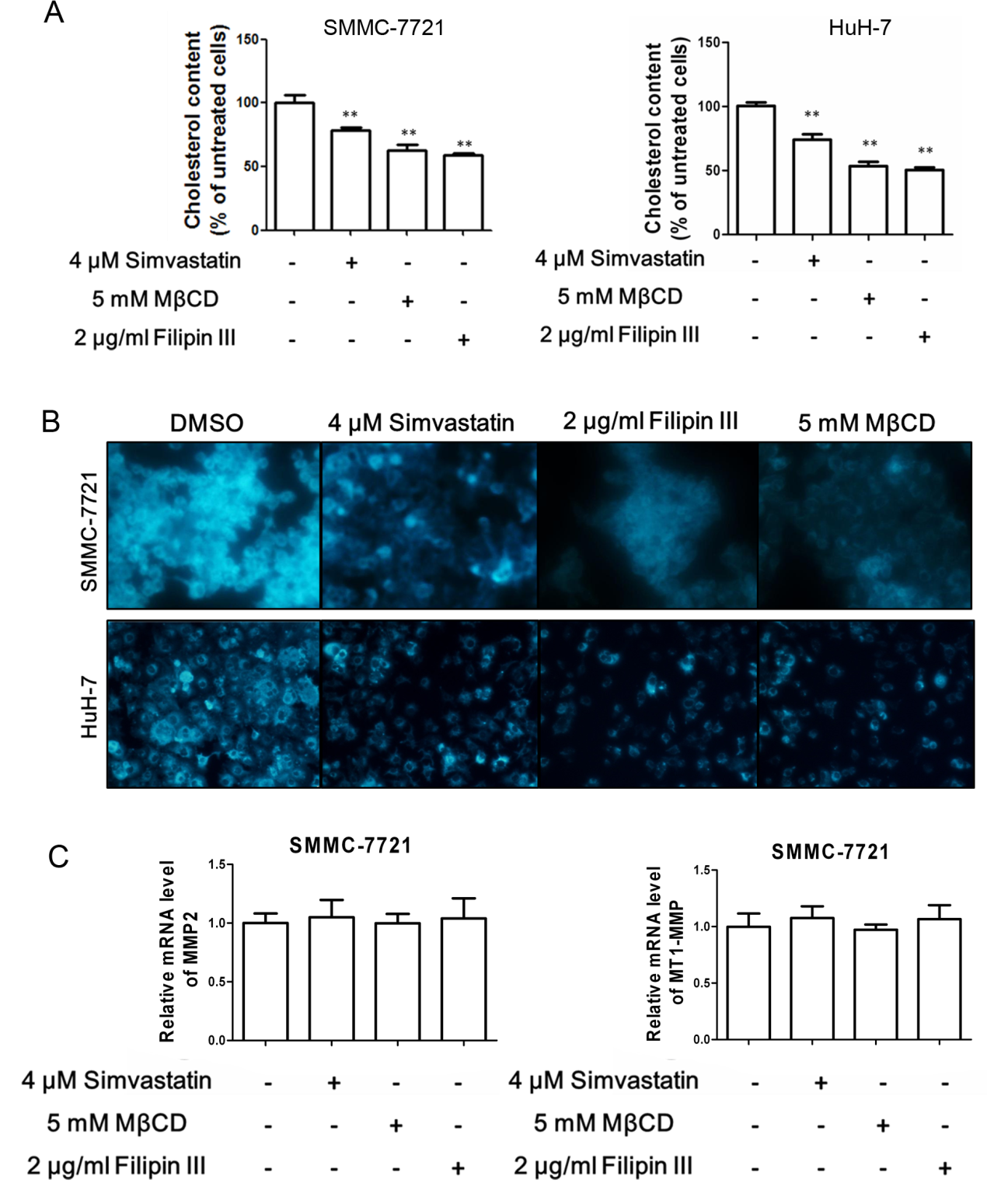


Fig. S2. **Cholesterol depletion mediated by simvastatin, MβCD or filipin III**. (A) Changes in the cellular cholesterol content of SMMC-7721 or Huh-7 cells after treatment with 4 μM simvastatin　(24 h), 5 mM MβCD (2 h) or 2 μg/mL filipin III (2 h). Cellular cholesterol content was assayed spectrophotometrically using an Amplex Red cholesterol assay kit (Invitrogen). **, *p*<0.01 compared with untreated cells. (B) Fluorescence micrographs of filipin staining by UV excitation. The membrane cholesterol of SMMC-7721 or HuH-7 cells (including cholesterol depleted samples) was detected by filipin staining. (C) Effects of cholesterol depletion on the mRNA levels of MMP-2 and MT1-MMP in SMMC-7221 cells.


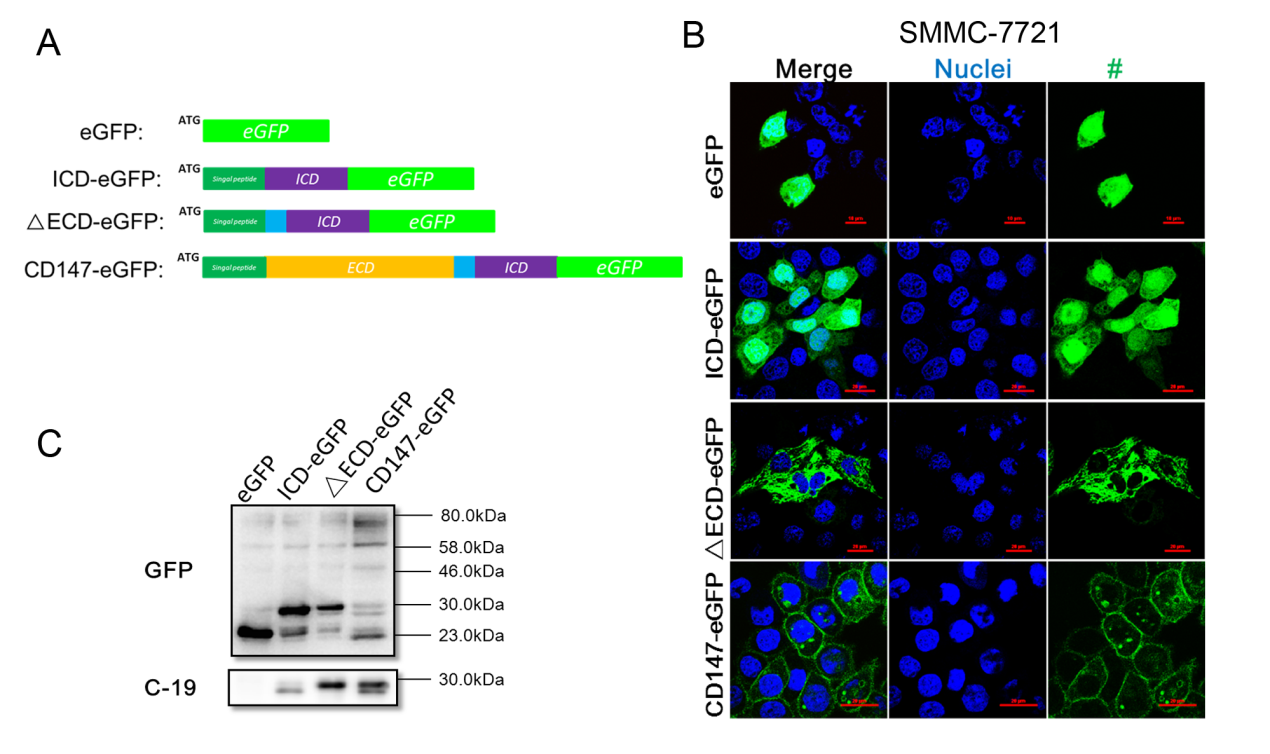


Fig. S3. **SMMC-7721/CD147-eGFP cells (SMMC-7721 cells stably expressing CD147-eGFP) intrinsically produced CD147-△ECD and CD147-ICD.** (A) Models of constructed plasmids with different parts of CD147 fused with eGFP. (B) The plasmids described above were transfected into SMMC-7721/eGFP-CD147 for 36 h. Then, the cytoplasm localization of different constructs was visualized with confocal microscopy. The nuclei were visualized by DAPI staining. Scale bar: 10 μm. (C) Cells transfected with separate constructs were collected and assessed via Western blot. c-19 is a commercial antibody recognizing C-terminal of CD147.


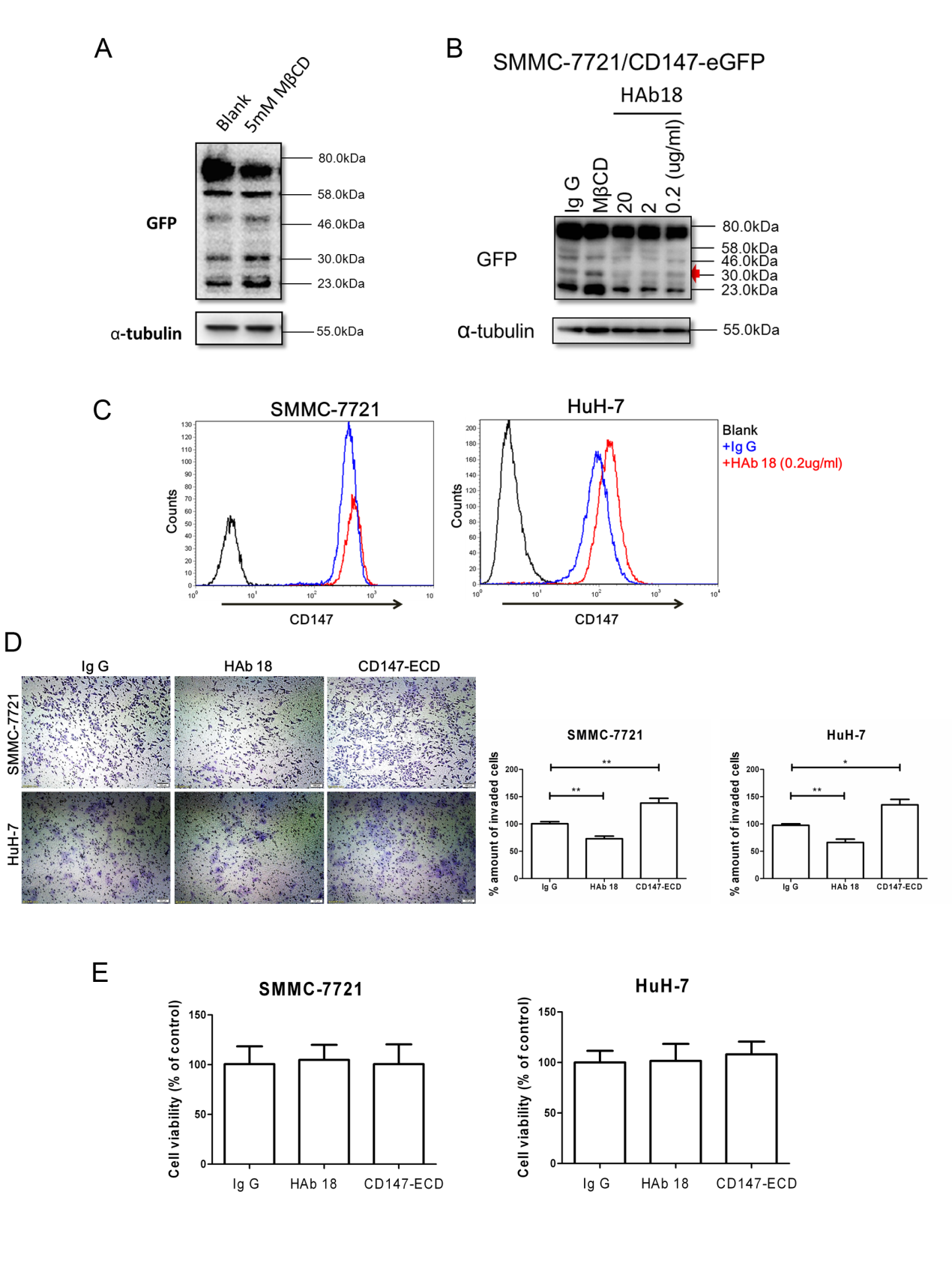


Fig. S4. **HAb 18 inhibits CD147 shedding and increases membrane CD147 levels but finally inhibits HCC cells invasion.** (A) Cleavage of CD147 caused by cholesterol depletion further produced CD147-ΔECD and CD147-ICD. SMMC-7721/CD147-GFP cells were administered 5 μM MβCD for 4 h, and then cells were collected and assessed via Western blot. (B) Growing SMMC-7721/CD147-GFP cells were first administered with different amounts of HAb 18 for 24 h and then assessed via Western blot. The truncated CD147 fused with GFP (as indicated by the arrowheads) was naturally produced. IgG: negative control; MβCD: positive control. (C) HAb 18 or IgG (1 μg/mL) was first added to growing SMMC-7721 or HuH-7 cells for 24 h. Then, cells were collected, incubated at 4 °C with secondary antibody in the dark for 30 min and analyzed with FACS. (D) HAb 18 inhibited the invasion of HCC cells. SMMC-7721 or HuH-7 cells were first collected and incubated with HAb 18 (1 μg/mL) or purified CD147-ECD (extracellular domain of soluble CD147 core protein, 10 μg/mL) for 30 min. Then, the invasive ability of HCC cells was measured via an *in vitro* invasion assay with a BD BioCoat Matrigel Invasion Chamber (BD, New Jersey, USA) (pore size, 8 µm). Left panel: representative image. Right panel: quantification. *, *p*<0.05, **, *p*<0.01. (E) Effects of HAb 18 or CD147-ECD on the cell growth of HCC cells. Growing SMMC-7721 or HuH-7 cells were treated with HAb 18 (0.1 μg/mL) or purified CD147-ECD (1 μg/mL) for 24 h, then the cell viability was assessed via MTT.


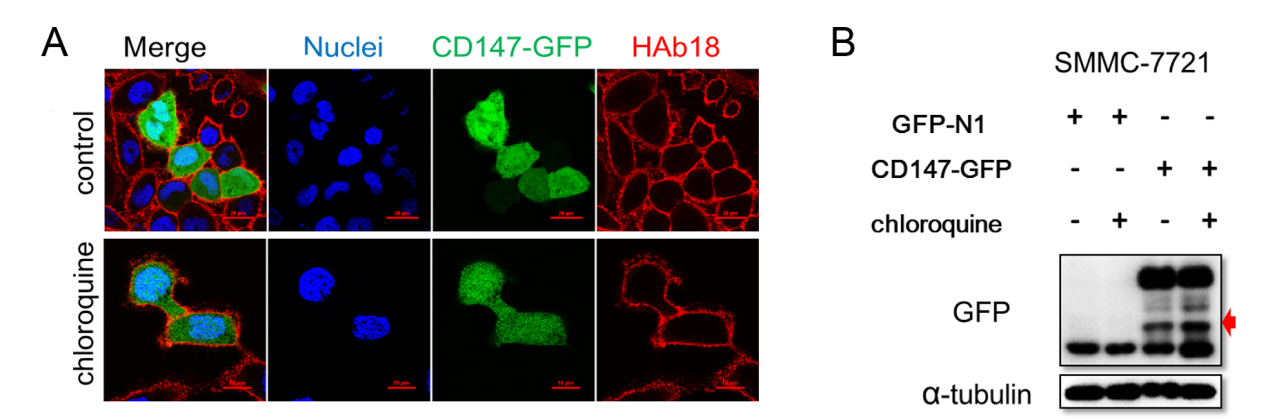


Fig. S5 (A) Immunofluorescence staining of CD147 with HAb 18 in SMMC-7721 cells transfected with e-GFP-N1 and then treated with/without chloroquine (20 μM). Bar, 20 µm. (B) Western blot analysis of GFP in the negative control group and the accumulated GFP fused with truncated CD147 (as indicated by the arrowheads) after treatment with chloroquine (20 μM) for 12 h.


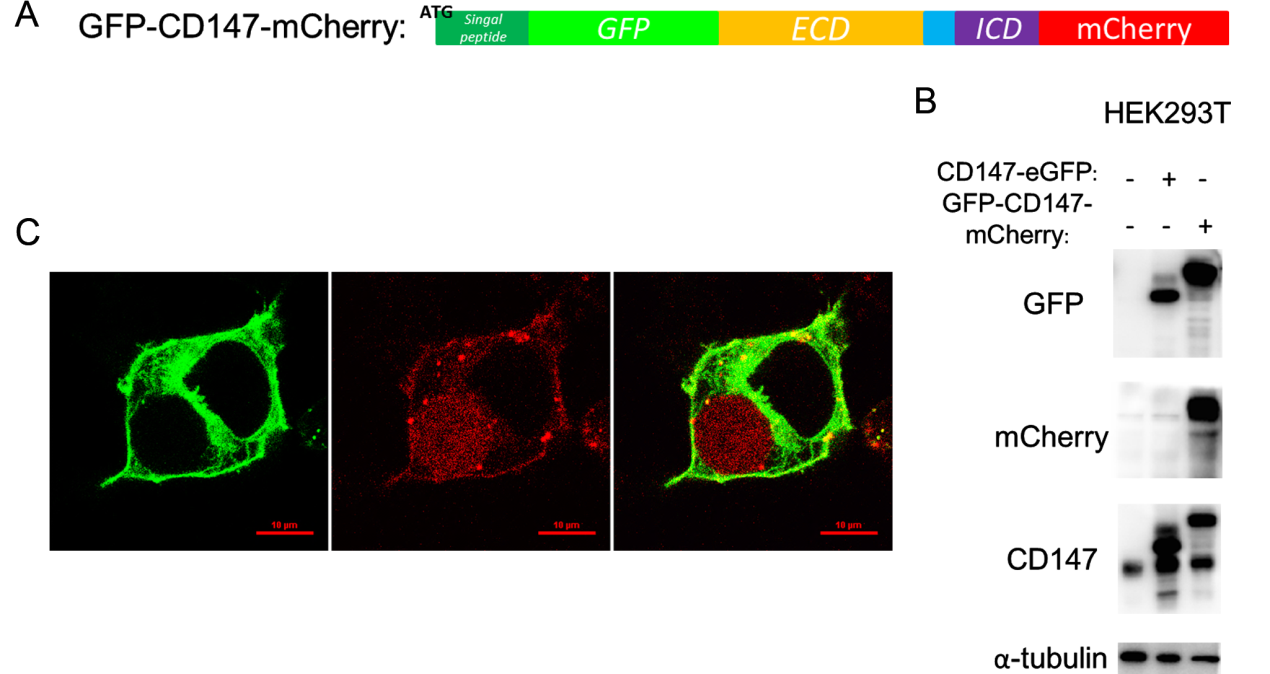


Fig. S6 **HEK293T cells transfected with GFP-CD147-mCherry exhibited nuclear-localized ICD-mCherry.** (A) A model of GFP-CD147-mCherry. (B) Western blot analysis of HEK293 cells transfected with GFP-CD147 or GFP-CD147-mCherry. (C) Representative image of HEK293 cells transfected with GFP-CD147-mCherry. Scale bar: 10 μm.


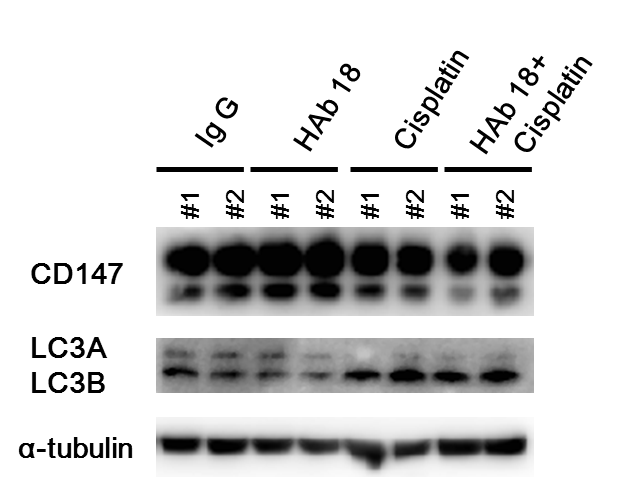


Fig. S7. Expression of CD147, LC3A/B in tumor tissues collected from mice groups with different treatment.
